# Identification of WD-Repeat Protein 72 as a Novel Prognostic Biomarker in Non-Small-Cell Lung Cancer

**DOI:** 10.1155/2023/2763168

**Published:** 2023-05-08

**Authors:** Guanglin Shi, Qinggan Ni, Yuqing Miao, Hua Huang, Zhongbo Yin, Weirong Shi, Minhua Shi

**Affiliations:** ^1^Department of Respiratory and Critical Care Medicine, Second Affiliated Hospital of Soochow University, Suzhou, Jiangsu 215004, China; ^2^Department of Respiratory Diseases, The Sixth People's Hospital of Nantong (Affiliated Nantong Hospital of Shanghai University), Nantong, Jiangsu 226011, China; ^3^Department of Burns and Plastic Surgery, Yancheng Clinical College of Xuzhou Medical University, The First People's Hospital of Yancheng, Yancheng 224000, China; ^4^Department of Pathology, Affiliated Hospital of Nantong University, Nantong, Jiangsu 226011, China; ^5^Department of Pathology, The Sixth People's Hospital of Nantong (Affiliated Nantong Hospital of Shanghai University), Nantong, Jiangsu 226011, China; ^6^Department of Thoracic Surgery, The Sixth People's Hospital of Nantong (Affiliated Nantong Hospital of Shanghai University), Nantong (Affiliated Nantong Hospital of Shanghai University), Jiangsu 226011, China

## Abstract

WD-repeat protein 72(WDR72; OMIM^∗^613214), a scaffolding protein lacking intrinsic enzymatic activity, produces numerous *β*-propeller blade formations, serves as a binding platform to assemble protein complexes and is critical for cell growth, differentiation, adhesion, and migration. Despite evidence supporting a basic role of WDR72 in the tumorigenesis of particular cancers, the value of WDR72 in non-small-cell lung cancer (NSCLC), the tumor with the highest mortality rate globally, is undocumented. We investigated the prognostic value of WDR72 in NSCLC and studied its potential immune function and its correlation with ferroptosis. According to The Cancer Genome Atlas, Cancer Cell Line Encyclopedia, Genotype-Tissue Expression, and Gene Set Cancer Analysis, we used multiple bioinformatic strategies to investigate the possible oncogenic role of WDR72, analyze WDR72 and prognosis, and immune cell infiltration in different tumors correlation. WDR72 exhibited a high expression in NSCLC and a positive association with prognosis. WDR72 expression was related to immune cell infiltration and tumor immune microenvironment in NSCLC. Finally, we validated WDR72 in human NSCLC; it has a predictive value in NSCLC related to its function in tumor progression and immunity. The significance of our study is that WDR72 can be used as a potential indicator of lung cancer prognosis. Helping physicians more accurately predict patient survival and risk of disease progression.

## 1. Introduction

Lung cancer is a deadly malignancy widespread worldwide, and non-small-cell lung cancer (NSCLC) represents approximately 85% of all lung cancers [1]. NSCLC onset is devious, with invisible clinical manifestations in the early stages, making early diagnosis hard. Furthermore, due to the highly aggressive nature of NSCLC, many patients have distant metastases at diagnosis [2]. Despite recent advances, early detection and treatment of NSCLC remain inadequate [3]. Gene mutation and aberrant expression lead to malignant alteration of airway epithelial cells, eventually leading to lung cancer [4]. Discovering new markers and driver genes is essential for the early detection, diagnosis, targeted treatment, and prognosis assessment of NSCLC.

WD-repeat protein 72(WDR72; OMIM^∗^613214) is a scaffold lacking intrinsic enzymatic activity. It produces several *β*-propeller blade formations, acting as binding platforms to assemble protein complexes and form tooth enamel, as well as causing autosomal recessive developmental defects [5]. Ibrahim-Verbaas et al. [6] identified a significant genome-wide association between a single nucleotide polymorphism (SNP) in the WDR72 gene (chromosome 15) and a cognitive test comparable to the Stroop interference test, demonstrating that at the level of executive function, WDR72 gene is also related to renal function. Howles et al. [7] demonstrated that WDR72 could involve in clathrin-mediated endocytosis, a critical process to persist intracellular CaSR signaling. WDR72 has a significant role in clear renal cell carcinoma [8], esophageal cancer [9], and colorectal cancer [10] but undetected in NSCLC. Accordingly, this paper intended to further analyze the specific mechanism of WDR72 and NSCLC.

Ferroptosis is a sort of controlled necrosis that performs a part in neurodegenerative cancers. Lipid peroxidation inhibitors, lipophilic antioxidants, and iron chelators can prevent ferroptosis [11]. Initially, ferroptosis was only recognized as erastin-induced death; however, additional studies revealed its function in various pathophysiological processes and diseases. Since certain oncogenically mutated tumor cells are more susceptible to ferroptosis, ferroptosis can be triggered, and ferroptosis-sensitive tumor cells may have significant therapeutic potential [12–14]. Therefore, modulation of ferroptosis may offer therapeutic potential for certain disorders associated with ferroptosis. Many key targets are important mediators of ferroptosis induction; however, the relationship between WDR72 and ferroptosis is still unclear. Herein, we further study whether WDR72 is sensitive to ferroptosis-induced cell death. Studies have shown that excessive iron overload may suppress the function of the immune system, thereby increasing the risk of infection and tumor development [15]. Immune infiltration refers to the presence of immune cells (T cells, B cells, macrophages, natural killer cells, etc.) in tumor tissues [16]. The immune infiltration of lung cancer patients is closely related to the survival and prognosis of patients [17]. Immune cells can slow the growth and spread of tumors by attacking tumor cells [18]. However, studies have also shown that tumor cells can evade the attack of immune cells through different mechanisms, thereby suppressing the immune response [19]. Overall, immune infiltration and ferroptosis are associated with the prognosis and survival of lung cancer patients, but more studies are needed to confirm the direct relationship between the two.

## 2. Materials and Methods

### 2.1. Gene Expression and Clinical Data Collection

First, we downloaded gene expression and clinical transcriptome profiling information for NSCLC patients from The Cancer Genome Atlas (TCGA) website (http://portal.gdc.cancer. gov/). The R tool “limma” was utilized to normalize raw gene expression information. GSE19804 [20, 21], GSE118370 [22], GSE19188 [23], GSE27262 [24, 25], and GSE33532 [26] were obtained from the GEO database GPL570 platform ([HG-U133_Plus_2] Affymetrix Human Genome U133 Plus 2.0 Array) through the “GEOquery” package for subsequent analysis (Table 1).

### 2.2. Identification DEGS

The selected datasets above were compared and examined utilizing the NCBI (https://www.ncbi.nlm.nih.gov/) toolGEO2R (http://www.ncbi.nlm.nih.gov/geo/geo2r). We applied adjusted (adj.) *p* values and thresholds to the Benjamini and Hochberg false discovery rates (FDR) are utilized to develop a balance between the statistical thresholds of finding significant genes and false positives. Probe sets without matching gene symbols or genes with several probe sets were excluded or averaged accordingly. Log fold change (FC) > 1.2 and adj < 0.01 indicated statistical significance.

### 2.3. Analysis of Association between WDR72 and Prognosis of Patients with Small-Cell Lung Cancer (SCLC)

In the TCGA database, a Kaplan-Meier (KM) analysis was conducted to examine the overall survival (OS), disease-specific survival (DSS), disease-free survival (DFS), and progression-free survival (PFI) of patients with NSCLC. To show the connection between WDR72 expression and survival in NSCLC, a Cox regression analysis was done utilizing the R tools “survival” and “forest plot.”

### 2.4. Association between WDR72 Expression and Immunity

Employing the ESTIMATE algorithm, immune and stromal scores of NSCLC samples were measured to investigate the association between WDR72 expression and TME (tumor microenvironment). The relationship between WDR72 expression and score was assessed utilizing the R tools “estimate” and “limma” based on the degree of immune infiltration. We downloaded the TCGA immune cell infiltration value from the CIBERSORT database (https://cibersortx.stanford.edu/) and calculated the relative score of 24 immune cells in NSCLC utilizing the CIBERSORT algorithm. Then, the Spearman rank correlation coefficient was employed to assess the association between the level of WDR72 and the infiltration level of each immune cell in NSCLC. R tools “limma,” “reshape2,” and “RColorBrewer” were utilized to present the visualizations.

### 2.5. Gene Set Enrichment Analysis

Gene Ontology (GO) and Kyoto Encyclopedia of Genes and Genomes (KEGG) gene sets were downloaded from the Gene Set Enrichment Analysis (GSEA) website. The biological role of WDR72 in NSCLC was investigated using the GSEA method. Both analyses were performed using the R “Cluster Profiler” tool.

### 2.6. Clinical Samples

We should be confirmed non-small-cell lung cancer at Nantong University from 2012 to 2020. A total of 158 matched NSCLC and paracancerous tissues were obtained through radical resection in the Affiliated Hospital of Nantong University. All diagnoses were confirmed by histopathological examination. For further study, the specimens were frozen in a -80°C freezer after surgery. All 158 patients had signed written informed consent and underwent a standardized ethnic review.

### 2.7. Reverse Transcription-Polymerase Chain Reaction (RT-qPCR)

Four matched lung adenocarcinoma and paracancerous tissues were randomly selected to extract total RNA. RNA was reverse transcript into cDNA utilizing a reverse transcription kit (transgene Biotech, China). NanoDrop 2000 (Thermo Fisher Scientific, Waltham, USA) was utilized to detect its fluorescent expression. GAPDH (glyceraldehyde-3-phosphate dehydrogenase) served as an internal control. WDR72 relative expression was computed employing the 2^-*ΔΔ*^CT approach.WDR72 F:GCAACTCAAACTCGGCAAACTTCC, R:GGCTCACCTGGACTCTCAGACTC.

### 2.8. Immunohistochemistry Staining Analysis

Paraffin-embedded NSCLC tissue sections were deparaffinized, hydrated, antigen retrieved, and goat serum blocked. Therefore, samples were incubated overnight at 4°C with an anti-WDR72 antibody (thermofisher PA5-63780 1 : 200) and species-specific secondary antibody for 30 min at 37°C. The immunosignal of the samples was observed utilizing a DAB solution and double-stained with hematoxylin in turn. A light microscope was used to obtain representative immunohistochemical (IHC) staining images.

### 2.9. Western Blot Analysis

Protein specimens from NSCLC and paracancerous tissue were isolated and transmitted to polyvinylidene fluoride (PVDF) membranes for the next steps. Following blocking with 5% BSA, PVDF membranes were incubated overnight at 4°C with primary antibodies to WDR72 (Thermo Fisher PA5-637801 : 2000) and GAPDH (1 : 1000) and then were incubated at room temperature (15-25°C) with HRP-labeled secondary antibodies for 1 h (1 : 1000) 2000). ECL (enhanced chemiluminescence) is used to capture images. GAPDH was chosen as the internal control.

### 2.10. Statistical Analyses

All data were normalized on gene expression using log2 transformation. Analyses of the correlation between two variables were carried out utilizing Spearman or Pearson test. *p* < 0.05 was judged statistically significant. Differences between adjacent tissues and cancerous tissues were conducted employing the Wilcox test. *p* < 0.05 was judged statistically significant. Kaplan-Meier curves and Cox proportional hazards regressions were employed for all survival analyses. R program (Ver. 4.1.2) was used to process the statistical significance of bioinformatic results, and GraphPad Prism (Ver. 9) was used to analyze the statistical importance of the in vitro data.

## 3. Results

### 3.1. Differential Expression of WDR72 between NSCLC Tumor and Normal Tissue Samples

**Figure 1:**
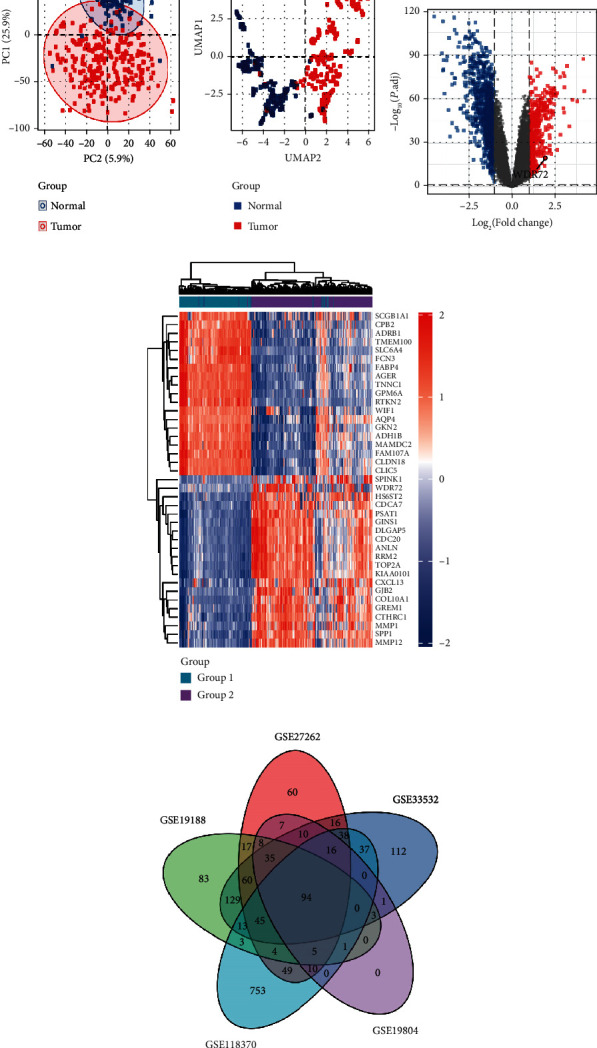
Five datasets of GPL570 platform in GEO database: GSE19804, GSE118370, GSE19188, GSE27262, and GSE33532. It can be seen from the PC graph and UMAP graph that the samples of the two groups are separated (a, b). Using the ggplot2 package of classical difference analysis-volcano plot to mark the location of WDR72 (c). (d) Visualizing the expression profile of the ComplexHeatmap package, the high and low expressions of each top 20 gene is shown. Venn diagram of (e) plots the crossover genes of the 5 datasets, showing that there are 94 crossover genes in the datasets.

**Figure 2:**
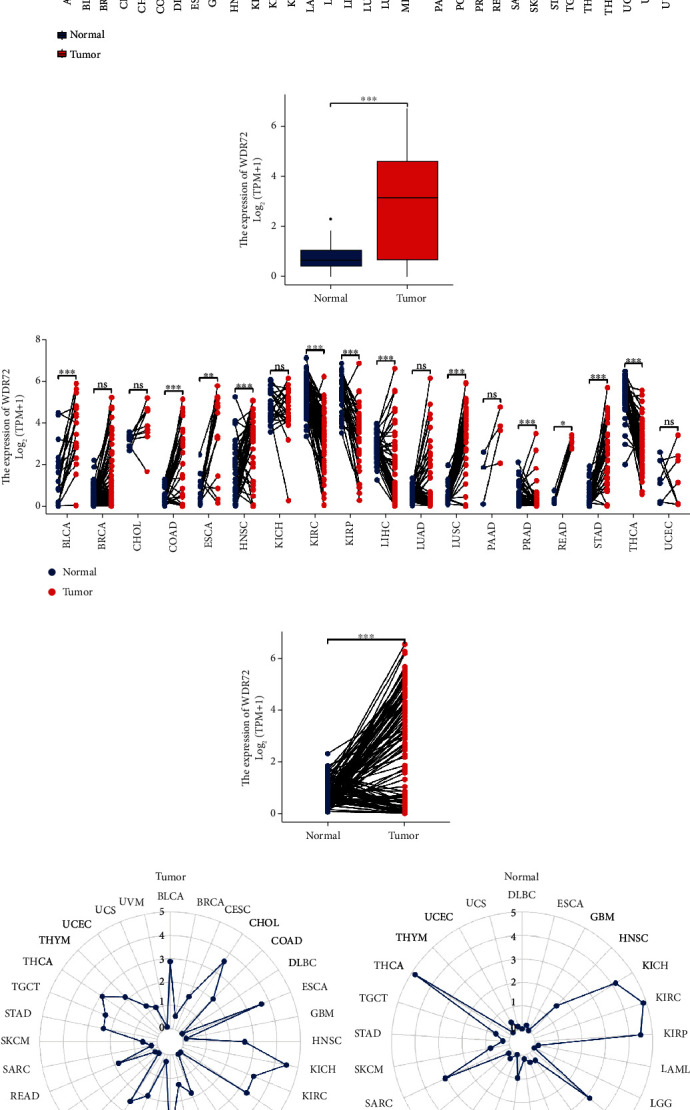
Differential expression of WDR72 in pan-cancer tissues and corresponding adjacent tissues and between paired and unpaired non-small-cell lung cancer tissues and corresponding adjacent tissues were analyzed using the package “ggplot2,” ns; *p* ≥ 0.05; ^∗^*p* ≤ 0.05; ^∗∗^*p* ≤ 0.01; ^∗∗∗^*p* ≤ 0.001 (a–d). Expression of WDR72 in pancreatic cancer tissues was analyzed using radar visualization in the “ggradar” and “ggplot2” software packages. (e) The expression of WDR72 in normal tissues adjacent to pan-cancer tumors (f).

We selected five datasets of GPL570 platform in GEO database, GSE19804, GSE118370, GSE19188, GSE27262, and GSE33532; “limma” R package; and “umap” R package. By drawing the PC and UMAP graphs, the samples of the two groups were separated, showing a significant variation between the two groups. The subsequent difference analysis may have more meaningful results (Figures 1(a) and 1(b)). Using the ggplot2 package of classical difference analysis-volcano plot, the threshold was selected as |logFC| ≥ 1.2, *p* value < 0.05. The figure is highlighted in blue or red, and the location of WDR72 is highlighted (Figure 1(c)).  Figure 1(d) depicts the visual expression profile of the ComplexHeatmap package and shows the expression of each top 20 genes with high and low expressions. Concurrently, the Venn diagram of Figure 1(e) plots the intersecting genes of the five datasets, showing 94 intersecting genes. Then, we evaluated the expression degrees of WDR72 in 33 cancers from TCGA data. The “ggplot2” package was utilized to examine the differential expression of WDR72 in pan-cancer tissues and corresponding adjacent tissues and the expression in paired and unpaired NSCLC tissues and corresponding adjacent tissues, ns, *p* ≥ 0.05, ^∗^*p* ≤ 0.05; ^∗∗^*p* ≤ 0.01; ^∗∗∗^*p* ≤ 0.001 (Figures 2(a)–2(d)). Radar visualization using the “ggradar” and “ggplot2” packages was utilized to identify WDR72 expression in pan-cancer tissues (Figure 2(e)) and adjacent pan-cancer tissues (Figure 2(f)). WDR72 was differentially expressed in 27 tumors except ACC, CESC, CHOL, DLBC, KICH, and PCPG.

### 3.2. Correlation Analysis of WDR72 with Clinical Factors in TCGA Database

**Figure 3:**
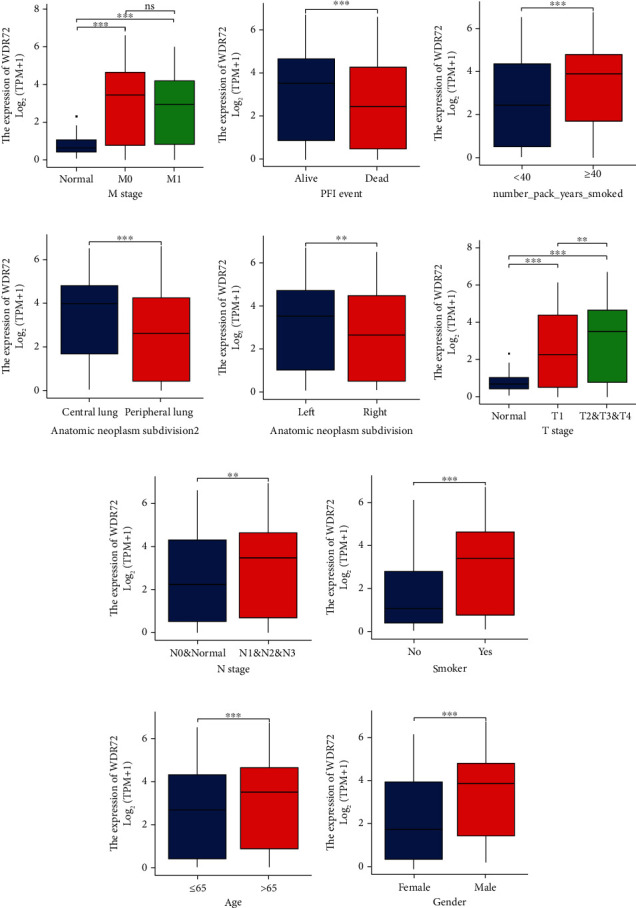
Correlation analysis between WDR72 and clinic. M grade correlation (a). PFI event correlation (b). Smoking year correlation (c). Tumor distribution (central or peripheral) correlation (d). Tumor distribution (left lung or right lung) correlation (e). T grade correlation (f). N grade correlation (g). Smoking correlation (h). Age correlation (i). Gender correlation (j). significance indicator: ns; *p* ≥ 0.05; ^∗^*p* ≤ 0.05; ^∗∗^*p* ≤ 0.01; ^∗∗∗^*p* ≤ 0.001.

**Figure 4:**
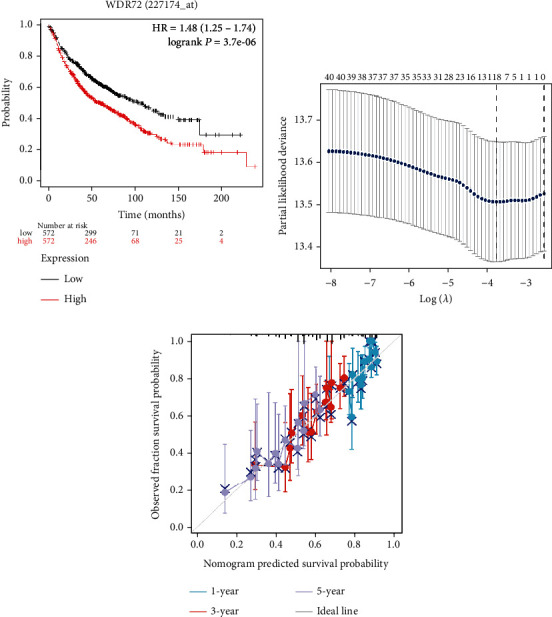
(a) Survival analysis of WDR72 non-small-cell lung cancer using the Kaplan-Meier plotter (http://kmplot.com/analysis). (b) The “glmnet” package was used to draw a LASSO variable trajectory was also drawn. (c) The calibration visualization. The abscissa is the survival probability predicted by the model, the ordinate is the actual observed survival probability, and the gray diagonal line is the ideal line.

RNAseq and clinical data in level 3 HTSeq-FPKM form in the TCGA (https://portal.gdc.cancer.gov/), LUAD (lungadenocarcinoma) and LUSC (lung squamous cell carcinoma) (lung cancer) experiments were excluded from control/normal (unapplied in projects without control/normal)+retained after clinical data availability. R package “ggplot” (Ver. 3.6.3) was used to evaluate the clinical correlations. Clinical analysis showed that the expression of WDR72 gene was related to whether smoking, age, lesion location, and TNM stage. M grade correlation is presented in Figure 3(a). PFI event correlation is presented in Figure 3(b). Smoking years correlation is presented in Figure 3(c). Tumor distribution (central or peripheral) correlation is presented in Figure 3(d). Tumor distribution (left lung or right lung) correlation is presented in Figure 3(e). T grade correlation is presented in Figure 3(f). N grade correlation is presented in Figure 3(g). Smoking correlation is presented in Figure 3(h). Age correlation is presented in Figure 3(i). Gender correlation is presented in Figure 3(j). Significance indicator: ns, *p* ≥ 0.05; ^∗^*p* ≤ 0.05; ^∗∗^*p* ≤ 0.01; ^∗∗∗^*p* ≤ 0.001; Figure 4(a) is the survival analysis of WDR72 in NSCLC on the Kaplan-Meier Plotter website (https://kmplot.com/analysis). The 5-year survival rate was significantly decreased for higher WDR72 expression patients compared to those with lower WDR72 expression. Control/normal (unapplied in projects without control/normal) was removed, and the clinical data was retained.  Figure 1(d) shows genes with higher expression differences in the heat map employing the R (ver. 3.6.3) “glmnet” tool (ver. 4.1-2) and survival tool (ver. 3.2-10) to draw the LASSO analysis utilizing the RMS tool (Ver. 6.2-0) and the survival package (Ver. 3.2-10) in R (ver. 3.6.3, Figure 4(b)). The COX statistical method incorporated TMN and WDR72 parameters to visualize the prognosis calibration analysis (Figure 4(c)).

### 3.3. PPI Network Map, GO/KEGG, and GSEA Enrichment Analysis of WDR72-Related Differential Genes

**Figure 5:**
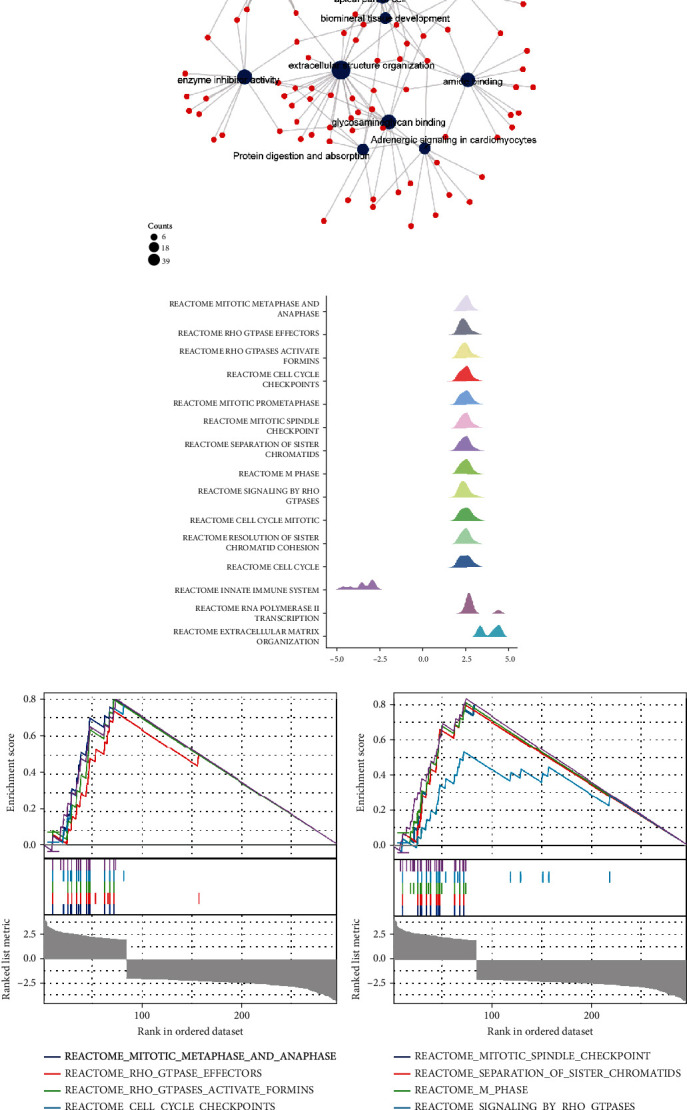
Data analysis of WDR72 differential genes (a, b) are, respectively, the GO/KEGG enrichment analysis diagram and protein interaction network diagram. (c) is the GSEA mountain map. (d–f) is the GSEA enrichment analysis map.

TCGA (https://portal.gdc.cancer.gov/) was utilized to obtain RNAseq information in level 3 HTSeq-Counts formed in the LUAD-LUSC (lung cancer) experiment after eliminating controls/normals (unapplied in projects without control/normal) using R (3.6.3 ver.) and “DESeq2” (ver. 1.26.0, Love MI et al. 2014) packages to target the molecule WDR72 [ENSG00000166415] in NSCLC by the reduce expression group: 0–50% and the increased expression group: 50–100% with analysis of differential genes of high correlation with WDR72. R (ver. 3.6.3) “ggplot2” tool (Ver. 3.3.3) was employed for visualization, and “clusterProfiler” package (Ver. 3.14.3) was used for WDR72 differential genes data analysis. (Figures 5(a) and 5(b)) show GO/KEGG enrichment analysis and protein interaction network, respectively.  Figure 5(c) depicts the GSEA mountain map, while Figures 5(d)–5(f) depict the GSEA enrichment analysis map. KEGG/GO and PPI analyses were shown in adrenergic signaling in cardiomyocytes, cytokine−cytokine receptor interaction, organelle fission, extracellular structure organization, biomineral tissue development, odontogenesis spindle, apical part of the cell, collagen−containing extracellular matrix, glycosaminoglycan binding, enzyme inhibitor activity, amide binding, receptor-ligand activity, protein digestion, absorption, and mitotic nuclear. GSEA enrichment analysis showed enrichment in reactome mitotic metaphase, anaphase, reactome RHO GTPase effectors, reactome RHO GTPases activate formins, reactome cell cycle checkpoints, reactome mitotic prometaphase, reactome mitotic spindle checkpoint, reactome separation of sister chromatids, reactome M phase, reactome signaling by RHO GTPases, reactome cell cycle mitotic, reactome resolution of sister chromatid cohesion, reactome cell cycle, reactome innate immune system, reactome rna polymerase II transcription, and reactome extracellular matrix organization.

### 3.4. Coexpression of WDR72 and Ferroptosis-Related Genes in NSCLC

**Figure 6:**
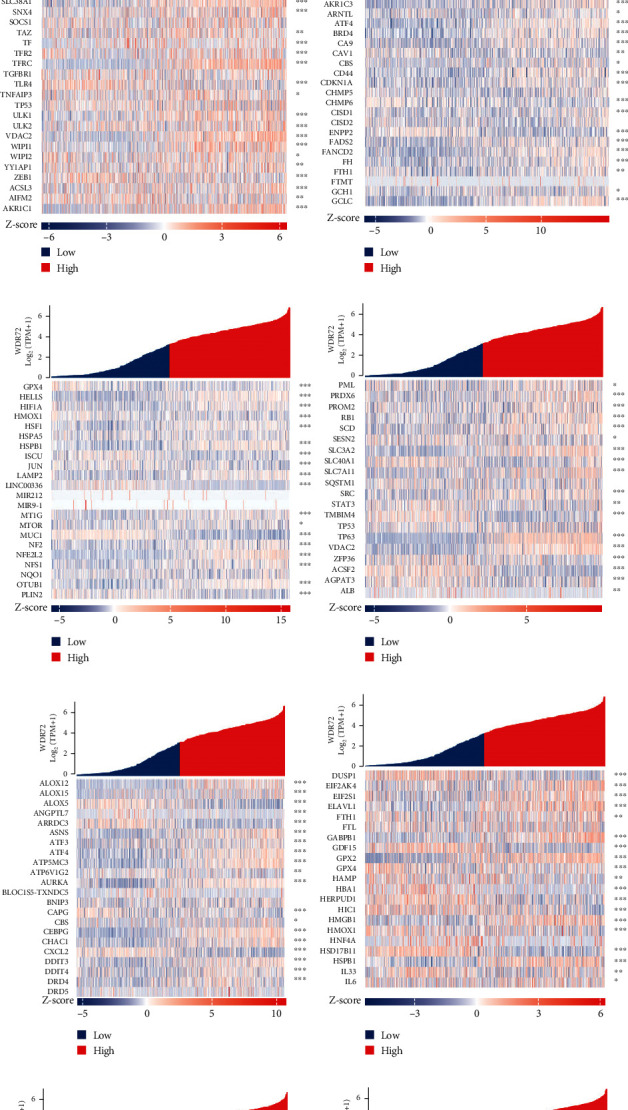
(a–m) TCGA (https://portal.gdc.cancer.gov/) removed target molecules in element LUADLUSC (lung cancer) and analyzed control/normal (not all elements have control/normal). Then use R (version 3.6.; 3) with the ggplot2 package: WDR72[ENSG00000166415] coexpression heat map of ferroptosis-related genes in non-small cell-lung cancer. Significance indicator: ns; *p* ≥ 0.05; ^∗^*p* < 0.05; ^∗∗^*p* < 0.01; ^∗∗∗^*p* < 0.001.

TCGA (https://portal.gdc.cancer.gov/) was utilized to acquire RNAseq data in level 3 HTSeq-FPKM form in the LUADLUSC (lung cancer) experiment after removing control/normal (unapplied in projects without control/normal) using R (ver. 3.6.3) in the “ggplot2” package to analyze the target molecule: WDR72 (ENSG00000166415) coexpression heat map of ferroptosis-related genes in NSCLC, significance indicator: ns, *p* ≥ 0.05; ^∗^*p* ≤ 0.05; ^∗∗^*p* ≤ 0.01; ^∗∗∗^*p* ≤ 0.001 (Figures 6(a)–6(m)).

### 3.5. Association of WDR72 with 24 Types of Immune Cells in NSCLC

**Figure 7:**
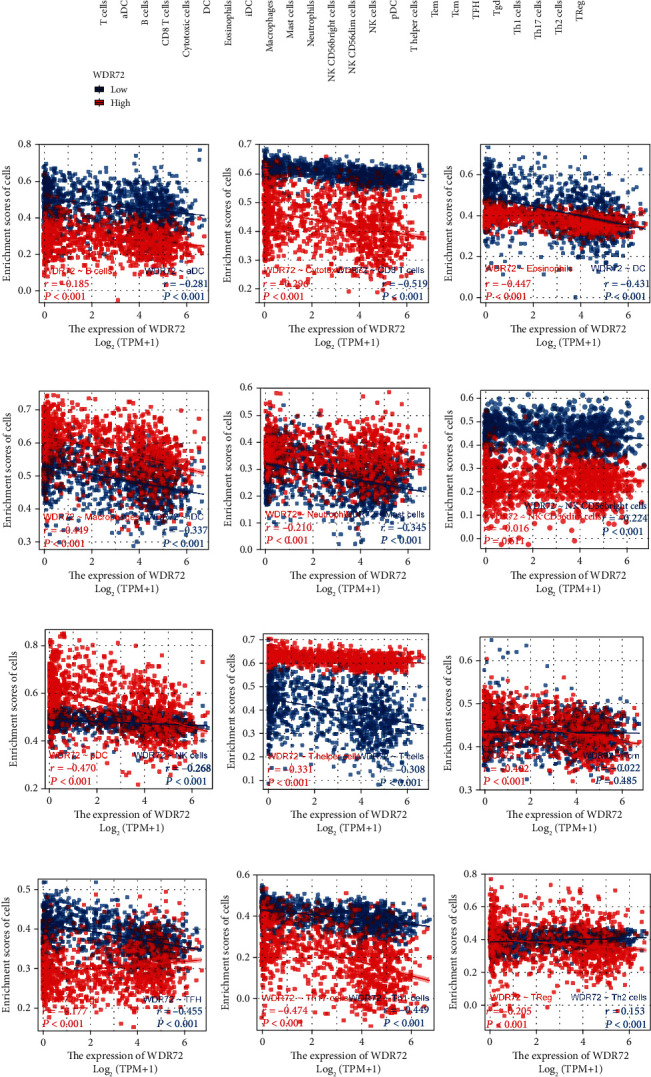
(a–m) TCGA (https://portal.gdc.cancer.gov/) RNAseq data in level 3 HTSeq-FPKM format in LUADLUSC (lung cancer) project after removing control/normal (not all projects have control/normal) using software: R (The GSVA package in version 3.6.3) has undergone the immune infiltration algorithm: ssGSEA (the built-in algorithm of the GSVA package) grouped WDR72 [ENSG00000166415] by the median and calculated the high and low expressions of WDR72 and 24 types of immune cells. Significant sign: ns; *p* ≥ 0.05; ^∗^*p* < 0.05; ^∗∗^*p* < 0.01; ^∗∗∗^*p* < 0.001.

It is well known that there are typical 24 types of immune infiltrating cells, namely, aDC cells, B cells, CD8 cells, T cells, cytotoxic cells, DC cells, eosinophils cells, iDC cells, macrophages cells, mast cells, neutrophils cells, NK CD56 bright cells, NK CD56dim cells, NK cells, pDC cells, T cells, T helper cells, Tcm cells, Tem cells, TFH cells, Tgd cells, Th1 cells, Th17 cells, Th2 cells, and TReg cells. TCGA (https://portal.gdc.cancer.gov/) was employed to recover RNAseq information in level 3 HTSeq-FPKM form in LUAD-LUSC (lung cancer) experiment after removing control/normal (unapplied in projects without control/normal) using R software (GSVA package ver. 3.6.3) to examine the immune infiltration method: ssGSEA (the built-in method of the GSVA) grouped WDR72 (ENSG00000166415) by the median and calculated the higher and lower expression of WDR72 and 24 forms of immunity cells, significant threshold: ns; *p* ≥ 0.05; ^∗^*p* ≤ 0.05; ^∗∗^*p* ≤ 0.01; ^∗∗∗^*p* ≤ 0.001. Except for Tcm and NK CD56, 24 immune cells had a nonsignificant correlation, while the *p* values of the other 22 cells were significant (Figures 7(a)–7(m)).

### 3.6. Clinical Correlation of WDR72 in NSCLC

**Figure 8:**
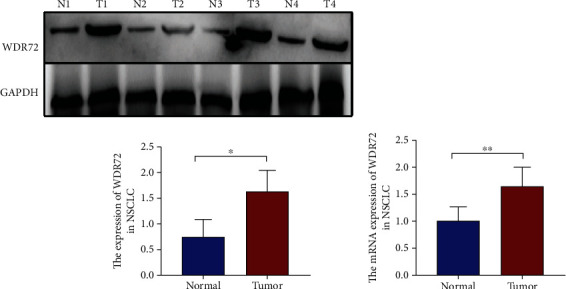
Expression of WDR72 in non-small-cell lung cancer (NSCLC). (a) Western blot analysis was used to detect the expression level of WDR72 in NSCLC tissues. (b) The expression level of WDR72 mRNA in NSCLC tissues was detected by qRT-PCR. ^∗^*p* < 0.05; ^∗∗^*p* < 0.01. N stands for normal tissue; T stands for tumor.

**Figure 9:**
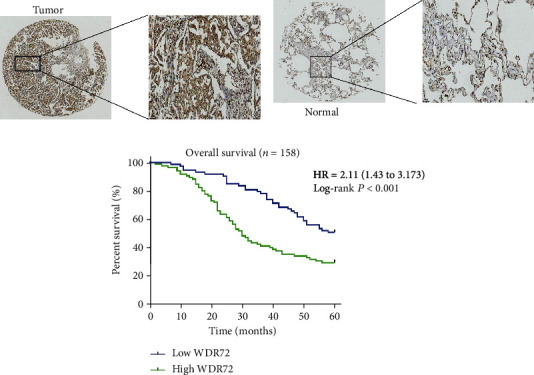
Expression of WDR72 in non-small-cell lung cancer (NSCLC). (a, b) Representative images of different IHC staining intensities of WDR72 in NSCLC tissues and corresponding adjacent normal tissues. (Tumor staining intensity score: 5)(Normal staining intensity score: 2) Magnification: x100 and x400. (c) Prognostic and survival analysis of 158 clinical NSCLC patients.

WDR72 was upregulated in NSCLC. To further validate the results of the previous analysis, we found the RNA and protein expressions of WDR72 in NSCLC. (Figures 8(a) and 8(b)) show a significantly greater level of WDR72 expression in NSCLC tissues compared to the matching normal tissues. Immunohistochemistry verified that WDR72 was strongly produced in NSCLC tissue proteins (Figures 9(a) and 9(b)). The results demonstrated that WDR72 expression level was greater in NSCLC tissues than in surrounding normal tissues. We collected clinical reports of 158 patients with NSCLC and discovered that patients with higher WDR72 levels had a poor prognosis (Figure 9(c)). Similarly, we found that WDR72 expression and smoking, tumor size, TNM staging, tumor grade, and metastasis were correlated (*p* < 0.05, Table 2). Multivariate analysis revealed that tumor size, TNM staging, tumor grade, and metastasis were associated with the expression of WDR72 (*p* < 0.05, Table 3).

## 4. Discussion

Lung cancer has two major histological forms: small-cell and non-small-cell. Genomic alterations have been found in LUSC and SCC. Lung cancer causes the greatest cancer-related deaths among males and females and inflammation and environmental risk factors such as smoking significantly contribute to lung cancer progression [27]. We analyzed the clinical significance of WDR72 in NSCLC data from the TCGA database WDR72. We discovered a close correlation with smoking, and the duration of smoking in years is also positively related to WDR72 expression. Concurrently, we used our clinical data to conduct univariate and multivariate analysis and found consistent results with the bioinformatic analysis; accordingly, WDR72 can be used as an NSCLC-target gene.

Ferroptosis was first identified as erastin-induced death and found to be involved in various pathophysiological processes and diseases. Since certain oncogenically mutated tumor cells have increased susceptibility to ferroptosis, ferroptosis can be triggered, and ferroptosis-sensitive tumor cells may also have significant therapeutic potential. Therefore, modulation of ferroptosis may have clinical potential in disorders related to ferroptosis [28]. The high-iron concentration of cancer cells and their increased susceptibility to developing ferroptosis are promising for cancer therapy [29]. Herein, we analyzed the correlation between WDR72 and ferroptosis-related genes using the R language and found that WDR72 was related to most ferroptosis-related genes. It is speculated that WDR72 may affect non-small-cells through the ferroptosis pathway of lung cancer progression.

TME is critical in cancer development, and different microenvironment signals have tumor-promoting and tumor-suppressing effects [30]. In TME, injury-related signals can influence the phenotype and activation condition of tumor cells and infiltrate immune cells [31]. The connection between the inflammatory microenvironment and tumors is bidirectional and dynamic and has both tumor-promoting and tumor-suppressing properties that it is not only the basis for the onset and progression of tumors [32]. Correlation analysis was performed in the NSCLC data, except for NK CD56dim cells and Tcm. In contrast, the other 22 cells were related, so we confirmed that WDR72 changes the TME through immune infiltration and impacts the progression of NSCLC.

Finally, our enrichment analysis indicated that WDR72 might be enriched through the cell cycle, immune system, RNA polymerase II transcription, and extracellular matrix organization. This suggested that it may affect tumor growth and metastasis through these pathways. Moreover, we conducted a series of tests to examine WDR72 expression in NSCLC tissues. WDR72 was elevated in NSCLC tissues relative to surrounding normal tissue.

Our study has some limitations. First, in vitro cell experiments are required to study WDR72 expression and function further. Second, our data suggest that WDR72 can act as a prognostic factor in NSCLC, which needs additional validation. Third, the presence of WDR72 on the TME and immunotherapy requires in vitro and in vivo experimental and clinical validation. Fourth, although we confirmed WDR72 expression in human NSCLC tissues, its exact regulatory mechanism remains unclear. WDR72 can participate in some biological processes in cells, including cell death, cell division, protein synthesis, and membrane transport. Ferroptosis is also a kind of programmed death. According to our research and analysis, WDR72 has a high correlation with many molecules of the ferroptosis pathway. Is it possible that WDR72 affects NSCLC through the ferroptosis pathway? In the next study, we will focus on the mechanism research.

## 5. Conclusion

Herein, the analysis of WDR72 suggested that WDR72 may be a prognostic factor in NSCLC, which was expressed at the mRNA and protein levels among NSCLC tumors and normal tissues. We initially revealed the association between WDR72 and ferroptosis and immune infiltration. Furthermore, WDR72 expression was linked to MSI, TMB, and immune cell infiltration in NSCLC. These findings may elucidate the aim of WDR72 in the incidence and growth of NSCLC and provide a reference for future patients with NSCLC to receive more accurate and personalized immunotherapy.

## Figures and Tables

**Table 1:** All data sources.

	Data sources	Website
1	TCGA	http://portal.gdc.cancer.gov/
2	NCBI	http://www.ncbi.nlm.nih.gov/
3	GEO2R	http://www.ncbi.nlm.nih.gov/geo/geo2r
4	CIBERSORT	https://cibersortx.stanford.edu/
5	GSE19804	https://www.ncbi.nlm.nih.gov/geo/query/acc.cgi?acc=GSE19804
6	GSE118370	https://www.ncbi.nlm.nih.gov/geo/query/acc.cgi?acc=GSE118370
7	GSE19188	https://www.ncbi.nlm.nih.gov/geo/query/acc.cgi?acc=GSE19188
8	GSE27262	https://www.ncbi.nlm.nih.gov/geo/query/acc.cgi?acc=GSE27262
9	GSE33532	https://www.ncbi.nlm.nih.gov/geo/query/acc.cgi?acc=GSE33532

**Table 2:** Relationship between WDR72 expression and clinicopathologic features of NSCLC patients.

Characteristics	Number	Expression of WDR72		*P* value^a^
		Low (*N* = 73)	High (*N* = 85)	
Age				0.425
≤60	72	36	36	
>60	86	37	49	
Gender				0.202
Female	77	40	37	
Male	81	33	48	
Smoke				0.025^∗^
No	75	42	33	
Yes	83	31	52	
Tumor size				0.038^∗^
≤3 cm	72	40	32	
>3 cm	86	33	53	
TNM stage				0.032^∗^
I and II	114	59	55	
III and IV	44	14	30	
Histology stage				0.037^∗^
Well	122	62	60	
Poorly	36	11	25	
Metastasis				0.016^∗^
Negative	85	47	38	
Positive	73	26	47	

^a^Chi-square test.^∗^*p* < 0.05.

**Table 3:** Univariate and multivariate analysis of prognostic factors of 5-year overall survival in WDR 72 patients.

Characteristic	Univariate analysis			Multivariate analysis		
	HR	95% CI	*P*	HR	95% CI	*P*
WDR72 expression						
Low vs. high	0.478	0.328~0.752	<0.001^∗∗^	0.624	0.404~0.963	0.033^∗^
Gender						
Male vs. female	0.706	0.471~1.057	0.091			
Age (years)						
≤60 vs. >60	0.925	0.617~1.386	0.705			
Smoking						
No vs. yes	0.605	0.402~0.910	0.016^∗^	0.885	0.546~1.432	0.618
Tumor size (cm)						
≤3 vs. >3	0.342	0.220~0.530	<0.001^∗∗^	0.827	0.495~1.381	0.468
TNM stage						
I/II vs. III/VI	0.219	0.144~0.332	<0.001^∗∗^	0.416	0.263~0.660	<0.001^∗∗^
Histology stage						
Well vs. poorly	0.356	0.232~0.548	<0.001^∗∗^	0.417	0.257~0.677	<0.001^∗∗^
Metastasis						
No vs. yes	0.192	0.124~0.298	<0.001^∗∗^	0.240	0.149~0.387	<0.001^∗∗^

HR: hazard ratio; CI: confidence interval; ^∗^*P* < 0.05; ^∗∗^*P* < 0.001.

## Data Availability

The utilized and examined datasets (Table 1) are available upon reasonable request from the corresponding author.

## References

[1] Amatu A., Sartore-Bianchi A., Bencardino K., Pizzutilo E. G., Tosi F., Siena S. (2019). Tropomyosin receptor kinase (TRK) biology and the role of *NTRK* gene fusions in cancer. *Annals of Oncology*.

[2] Jeon Y. J., Kim T., Park D. (2018). miRNA-mediated TUSC3 deficiency enhances UPR and ERAD to promote metastatic potential of NSCLC. *Nature Communications*.

[3] Schwaederlé M. C., Patel S. P., Husain H. (2017). Utility of genomic assessment of blood-derived circulating tumor DNA (ctDNA) in patients with advanced lung adenocarcinoma. *Clinical Cancer Research*.

[4] Koo T., Yoon A. R., Cho H. Y., Bae S., Yun C. O., Kim J. S. (2017). Selective disruption of an oncogenic mutant allele by CRISPR/Cas9 induces efficient tumor regression. *Nucleic Acids Research*.

[5] Zhang H., Koruyucu M., Seymen F. (2019). WDR72 mutations associated with amelogenesis imperfecta and acidosis. *Journal of Dental Research*.

[6] Ibrahim-Verbaas C. A., Bressler J., Debette S. (2016). GWAS for executive function and processing speed suggests involvement of the *CADM2* gene. *Molecular Psychiatry*.

[7] Howles S. A., Wiberg A., Goldsworthy M. (2019). Genetic variants of calcium and vitamin D metabolism in kidney stone disease. *Nature Communications*.

[8] Wu Y., Zhang X., Wei X. (2021). Development of an individualized ubiquitin prognostic signature for clear cell renal cell carcinoma. *Frontiers in Cell and Development Biology*.

[9] Warnecke-Eberz U., Metzger R., Hölscher A. H., Drebber U., Bollschweiler E. (2016). Diagnostic marker signature for esophageal cancer from transcriptome analysis. *Tumour Biology*.

[10] Zhang Z., Wang Q., Zhang M. (2021). Comprehensive analysis of the transcriptome-wide m6A methylome in colorectal cancer by MeRIP sequencing. *Epigenetics*.

[11] Cao L., Mu W. (2021). Necrostatin-1 and necroptosis inhibition: pathophysiology and therapeutic implications. *Pharmacological Research*.

[12] Chen P. H., Wu J., Ding C. C. (2020). Kinome screen of ferroptosis reveals a novel role of ATM in regulating iron metabolism. *Cell Death and Differentiation*.

[13] Zou Y., Palte M. J., Deik A. A. (2019). A GPX4-dependent cancer cell state underlies the clear-cell morphology and confers sensitivity to ferroptosis. *Nature Communications*.

[14] Sui X., Zhang R., Liu S. (2018). RSL3 drives ferroptosis through GPX4 inactivation and ROS production in colorectal cancer. *Frontiers in Pharmacology*.

[15] Stefanova D., Raychev A., Arezes J. (2017). Endogenous hepcidin and its agonist mediate resistance to selected infections by clearing non-transferrin-bound iron. *Blood*.

[16] Sa J. K., Kim S. H., Lee J. K. (2019). Identification of genomic and molecular traits that present therapeutic vulnerability to HGF-targeted therapy in glioblastoma. *Neuro-Oncology*.

[17] Kadara H., Choi M., Zhang J. (2017). Whole-exome sequencing and immune profiling of early-stage lung adenocarcinoma with fully annotated clinical follow-up. *Annals of Oncology*.

[18] Nagarsheth N., Wicha M. S., Zou W. (2017). Chemokines in the cancer microenvironment and their relevance in cancer immunotherapy. *Nature Reviews Immunology*.

[19] Poggio M., Hu T., Pai C. C. (2019). Suppression of Exosomal PD-L1 induces systemic anti-tumor immunity and memory. *Cell*.

[20] Lu T. P., Tsai M. H., Lee J. M. (2010). Identification of a novel Biomarker,SEMA5A, for non-small cell lung carcinoma in nonsmoking women. *Cancer Epidemiology, Biomarkers & Prevention*.

[21] Lu T.-P., Hsiao C. K., Lai L.-C. (2015). Identification of regulatory SNPs associated with genetic modifications in lung adenocarcinoma. *BMC Research Notes*.

[22] Xu L., Lu C., Huang Y. (2018). SPINK1 promotes cell growth and metastasis of lung adenocarcinoma and acts as a novel prognostic biomarker. *BMB Reports*.

[23] Hou J., Aerts J., den Hamer B. (2010). Gene expression-based classification of non-small cell lung carcinomas and survival prediction. *PLoS One*.

[24] Wei T. Y., Juan C. C., Hisa J. Y. (2012). Protein arginine methyltransferase 5 is a potential oncoprotein that upregulates G1 cyclins/cyclin-dependent kinases and the phosphoinositide 3-kinase/AKT signaling cascade. *Cancer Science*.

[25] Wei T. Y., Hsia J. Y., Chiu S. C. (2014). Methylosome protein 50 promotes androgen- and estrogen-independent tumorigenesis. *Cellular Signalling*.

[26] Quek K., Li J., Estecio M. (2017). DNA methylation intratumor heterogeneity in localized lung adenocarcinomas. *Oncotarget*.

[27] O’Brien T. D., Jia P., Caporaso N. E., Landi M. T., Zhao Z. (2018). Weak sharing of genetic association signals in three lung cancer subtypes: evidence at the SNP, gene, regulation, and pathway levels. *Genome Medicine*.

[28] Li J., Cao F., Yin H. L. (2020). Ferroptosis: past, present and future. *Cell Death & Disease*.

[29] Yee P. P., Wei Y., Kim S. Y. (2020). Neutrophil-induced ferroptosis promotes tumor necrosis in glioblastoma progression. *Nature Communications*.

[30] Iriondo O., Liu Y., Lee G. (2018). TAK1 mediates microenvironment-triggered autocrine signals and promotes triple-negative breast cancer lung metastasis. *Nature Communications*.

[31] Alvarado A. G., Thiagarajan P. S., Mulkearns-Hubert E. E. (2017). Glioblastoma cancer stem cells evade Innate immune suppression of self-renewal through reduced TLR4 expression. *Cell Stem Cell*.

[32] Pavlova N. N., Zhu J., Thompson C. B. (2022). The hallmarks of cancer metabolism: still emerging. *Cell Metabolism*.

